# Current Progress in Conductive Hydrogels and Their Applications in Wearable Bioelectronics and Therapeutics

**DOI:** 10.3390/mi14051005

**Published:** 2023-05-06

**Authors:** Bangul Khan, Saad Abdullah, Samiullah Khan

**Affiliations:** 1 Hong Kong Center for Cerebro-Cardiovascular Health Engineering (COCHE), Hong Kong SAR, China; balochbangulkhan@gmail.com; 2 School of Innovation, Design and Engineering, Division of Intelligent Future Technologies, Mälardalen University, P.O. Box 883, 721 26 Västerås, Sweden; 3 Center for Eye & Vision Research, 17W Science Park, Hong Kong SAR, China; dr.samipharmacist99@gmail.com

**Keywords:** conductive materials, wearable electronics, bioelectronics, sensing, drug delivery, smart hydrogels, biomaterials, biotherapeutics

## Abstract

Wearable bioelectronics and therapeutics are a rapidly evolving area of research, with researchers exploring new materials that offer greater flexibility and sophistication. Conductive hydrogels have emerged as a promising material due to their tunable electrical properties, flexible mechanical properties, high elasticity, stretchability, excellent biocompatibility, and responsiveness to stimuli. This review presents an overview of recent breakthroughs in conductive hydrogels, including their materials, classification, and applications. By providing a comprehensive review of current research, this paper aims to equip researchers with a deeper understanding of conductive hydrogels and inspire new design approaches for various healthcare applications.

## 1. Introduction

Wearable bioelectronics devices are currently dominating the healthcare sector because they offer many advantages over traditional biomedical devices, such as the ability to monitor physiological parameters within a person’s body without the assistance of professionals [1]. Traditional biomedical devices have numerous disadvantages, such as low accuracy, complex handling, time consumption, and storage issues. The use of conductive materials in wearable bioelectronic devices has captured the world’s attention and gained the community’s trust in using wearable bioelectronic devices instead of traditional biomedical devices [2].

Hydrogels are potentially biocompatible polymeric materials with a three-dimensional network [3], making them a more promising material in bioengineering. It has a high degree of flexibility, tunable mechanical properties, a high degree of hydrophilicity, and a greater swelling capability. It comprises natural and synthetic polymers, and each polymer is used differently depending on the hydrogel’s application [4]. For several decades, scientists have been making advances in hydrogels, and they have recently introduced smart hydrogels that are sensitive to external stimuli such as temperature, pressure, force, pH, and biological signals [5].

Conductive hydrogels are being investigated as a potential material for wearable bioelectronic devices [6]. These hydrogels have the same properties as traditional hydrogels but are also electrically conductive [7]. The polymeric materials in conductive hydrogels determine their electrical properties [8]. Several studies [9,10] have been conducted to improve the electrical properties of conductive hydrogel by adding carbon nanotubes, electrolytes, ionized liquids, graphene, and inorganic conductive filler to make them more suitable for wearable bioelectronic devices.

The conductive hydrogels were divided into three groups by the researchers based on their conductive components: ionic conductive hydrogels are made up of ionic liquid and electrolytes [11,12]; nanocomposite conductive hydrogels are made up of ionized nanotubes and inorganic conductive fillers, while polymeric conductive hydrogels are made up of conductive polymers [13]. The conductivity mechanism in conductive hydrogel works in two ways. In the first type of mechanism, ionic conductive materials are introduced into a three-dimensional network of polymers. Existing ions are migrated due to the attraction and repulsion of the introduced ions and hydrogel experienced conductivity [14]. The second mechanism integrates a conductive electronic component with a three-dimensional network of polymers to create an electron transport network that enables hydrogel conductivity [15]. Because of these mechanisms, conductive hydrogels have high electrical conductivity, tunable mechanical properties, and stimuli-responsive behaviors, making them a promising candidate for health monitoring and bioelectronic devices [16].

This review focuses on the most recent breakthroughs in conductive hydrogels and their applications in smart wearable bioelectronic devices. In the beginning, this review article highlighted the type of conductive materials and the classification of conductive hydrogel. While later sections discuss the investigated potential application of conductive hydrogels in wearable bioelectronic devices and therapeutics. This review paper will provide researchers with a thorough understanding of conductive hydrogels for wearable bioelectronics and a precise summary of various works, allowing them to make significant advances in designing new conductive materials for various healthcare applications.

## 2. Conductive Materials

### 2.1. Conductive Polymers

Conducting polymers are organic macromolecules that have electrical conductivity due to the polymers’ 3-D electronic network channel mechanism. Commonly conductive polymers used in the wearable bioelectronics include poly(pyrrole) (PPY), poly(aniline) (PANI), poly-(3,4-ethylenedioxythiophene) (PEDOT), polyacetylene (PAT), polythiophene (PTH) and poly(p-phenylene vinylene) (PPV), etc. [17].

Chalmers et al. recently published a study in which they improved the adhesion and conductive properties of a polypyrrole-based conductive hydrogel for wearable devices, concluding that electro-polymerization of polydopamine within the polypyrrole-based hydrogel can increase the conductivity and adhesion by (2720%) and (2140%), respectively, when compared to the unmodified PPY hydrogel [18].

**Figure 1:**
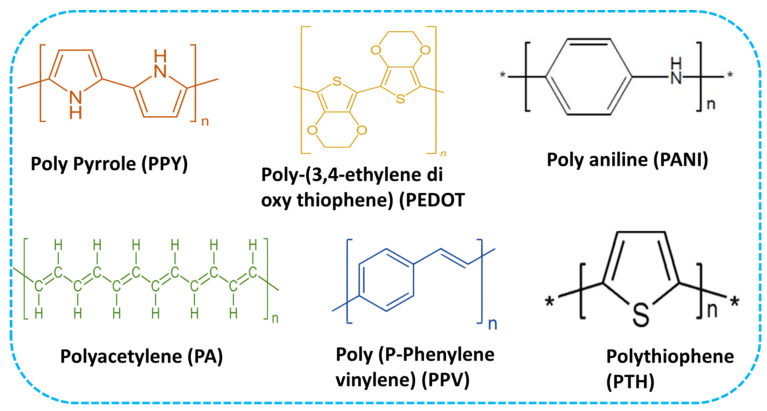
Chemical Structure of Conductive Polymers. Where star represents the repeat units.

Beygisangchin et al. extensively reviewed PANI, stating that it is the second most used conductive polymer for electrosensitive hydrogels after PPY. Many researchers were pulled to PANI because of its potential properties, such as high sensitivity, reversible doping, dead doping, low expenditure, simple synthesis, and mechanical stability [19]. Nie et al. reported a mini review on PEDOT. Authors highlighted that PDOT could be a potential candidate for wearable electronic devices due to their unique properties and the fact that they are already used in energy conversion, sensing, and storage applications. Additionally, they have high conductivity, flexible mechanical property, strong chemical stability, easy doping, and good optical transparency [20]. Furthermore, the chemical structure of conductive polymers is highlighted in Figure 1, and the summarized overview of the conductive polymers are listed in Table 1.

### 2.2. Metal Nanoparticles

Polymeric-based conductive hydrogels experienced limitations in soft robotics and ultra-sensitive applications due to the demand for ultra-conductivity and sensitivity. Researchers aim to modify the hydrogel by using metallic nanoparticles to overcome the shortcomings of conductive hydrogels [26].

Potential metallic nanoparticles for use in conductive hydrogels include platinum nanoparticles (Pt NPs), gold nanoparticles (Au NPs), silver nanoparticles (Ag NPs), and palladium nanoparticles (Pd NPs). The summarized properties of the metallic nano particles are listed in Table 2 [27].

**Figure 2:**
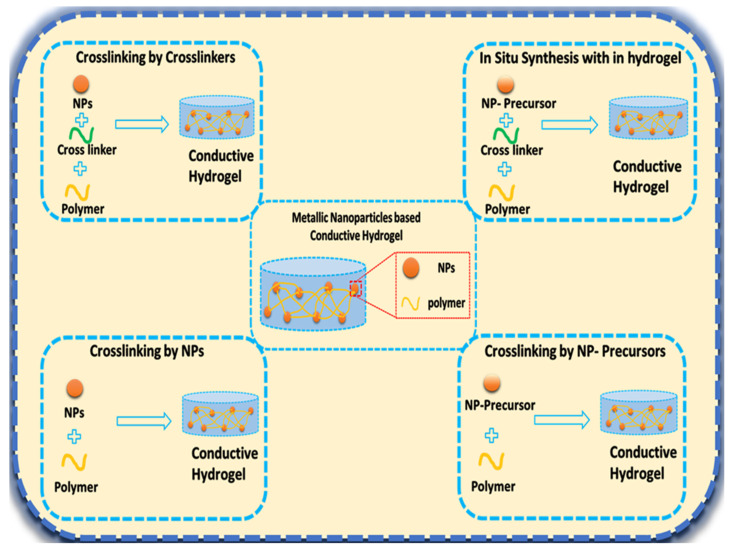
Crosslinking approaches of the metal nanoparticles with the polymer’s monomers.

Crosslinking is a crucial stage in the formulation of conductive hydrogels. Figure 2 illustrates four methods proposed in the literature for crosslinking metallic nanoparticles with the polymeric matrix. The first method utilizes a crosslinker to crosslink the nanoparticles, whereas the second employs NP precursors instead of the nanoparticles. In the third method, nanoparticles directly crosslinked with polymers without the assistance of a crosslinker. Furthermore, the final approach uses the NPs precursors for direct crosslinking [28].

**Table 2:** Properties of Metallic Nanoparticles.

Metallic NPs	Diameter (nm)	Density (g/cm^3^)	Melting Point (°C)	Boiling Point (°C)	Conductivity (S·cm^−1^)	Advantages	Limitations	Applications
Pt NPs [29]	~1.2	~21.45	~1772	~3827	~0.09	High stability and conductivity	Cytotoxicity and high price	Biosensing and tumor detection
Au NPs [30]	~9.1	~19.30	~1064	~2807	0.3~0.8	High stability and low toxicity	High price and low optical properties	Drug delivery, biosensing, and tumor cell treatment
Pd NPs [31]	~3.8–5.2	~12.02	~1555	~2970	~0.06	High stability and high optical properties	Cytotoxicity and low sensitivity	Biosensing and actuators
Ag NPs [32]	~12–30	~10.5	~961.78	~2162	0.5~0.7	High optical properties and antimicrobial	Cytotoxicity and high price	Antimicrobial, biosensing, and transdermal drug delivery

### 2.3. Carbons

**Figure 3:**
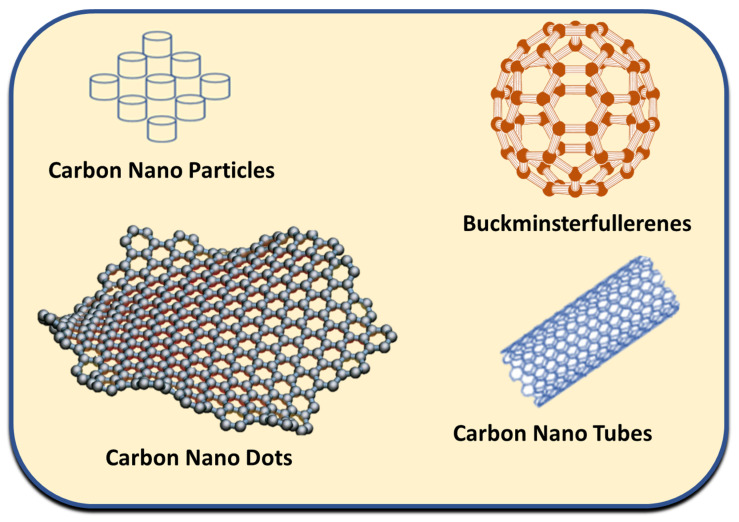
Visual structure of carbon derivatives.

Carbon is a naturally occurring element with strong electrical conductivity due to its four valence electrons. Moreover, the arrangement of carbon atoms results in the conductivity of the different materials, such as in carbon nanotubes and graphite nano tubes the parallel arrangement of carbon atoms result in their high conductivity while graphite is low conductor due to the perpendicular arrangement of carbon atoms in the plane. In parallel arrangements, carbon atoms move freely between layers and due to their four-valance electron movement electrical conductivity is experienced. It is extensively utilized in soft conductive materials due to its high sensitivity, electrical conductivity, excellent biocompatibility, flexible mechanical characteristics, and exceptional doping properties [33]. The potential derivatives of carbons include carbon nanotubes, carbon nanoparticles, and carbon dots, which are further shown and summarized in Figure 3 and Table 3.

Carbon based conductive hydrogels and soft materials offer a wide range of properties, such as enhanced electrical conductivity, high toughness, good adhesiveness, self-healing, stretchability, flexible mechanical properties, and strong chemical properties. they can be used in a wide variety of applications, including biosensing, wearable electronics, and drug delivery applications [37], which is discussed in the last section of the paper. Carbon-based materials are excellent candidates for soft conductive materials for conductive hydrogels to advance wearable bioelectronics.

### 2.4. Hybrid Materials

In biomaterials, hybrid materials were introduced in the last few decades, in which the combination of two different materials improved the required property. So, in the context of conductive hydrogels, the researchers investigated different material combinations to overcome the issues of hydrophobicity and mechanical strength [37].

Currently, the researchers investigated the combination of natural polysaccharide, cellulose, hemicellulose, poly vinyl alcohol, polypyrrole, poly aniline, alginate, PEDOT, polyacetylene, polythiophene to tune the physiochemical and mechanical properties of the conductive hydrogels [38]. Ren et al. investigated hybrid conductive hydrogel for electrochemical sensors and bioelectronics. The hybrid conductive hydrogel contains polypyrrole and PEDOT: PSS conductive materials and is prepared via a simple solution mixing method. The results demonstrated the enhanced electrical conductivity of 867 S·m^−1^ with good biocompatibility and mechanical strength. Additionally, the investigated hybrid hydrogel offered a real-time monitoring of cell proliferation and biomolecular detection [39]. Sun et al. reported the hybrid conductive hydrogel for ultra-conductivity and stretchability, which contains poly acrylamide and PEDOT, PSS as conductive components. The results demonstrated successful crosslinking with an enhanced sensitivity range of 0–2850% strain with a response time of 200 m·s [40]. Lovely et al. reported a polymeric electroconductive composite synthesized from protein nanowires. The material was formulated by the microorganism Geobacter sulfurreducens, which dispersed nanowires in a polymeric matrix. The reported innovation claims high conductivity of biosensors and wearable electronic devices by using this material [41]. Li et al. reported an innovative method to synthesize the ionic conductive hydrogel using hybrid materials containing Polyacrylic-Fe^3+/^ silver. The reported work claimed high mechanical strength with extensive stretchability and conductivity. It can be used as a promising material to solve the dual problems of conductivity and mechanical characteristics [42]. Yadavalli et al. invented the supercapacitor system using hybrid conductive polymers instead of metals or organic solvents. The supercapacitor system claimed to be biodegradable and biocompatible, contained a flexible protein substrate, conductive ink, and gel electrolytes. The reported system can be potentially investigated to replace the toxic metallic material with biocompatible conductive hybrid polymers [43]. Furthermore, the summarized characteristics are listed in Table 4.

## 3. Classification of Conductive Hydrogels

### 3.1. Ionic Conductive Hydrogels

Ionic Conductive hydrogel contains repeating cationic and anionic groups in a three-dimensional network with holes through which ions can easily travel to create conductivity inside the hydrogel network and synthesized by ionizing saline solutions with poly electrolytes. The researchers reported various studies on the ionic conductive hydrogel but failed to achieve the desired properties, such as biocompatibility, self-healing, and transparency [45].

**Figure 4:**
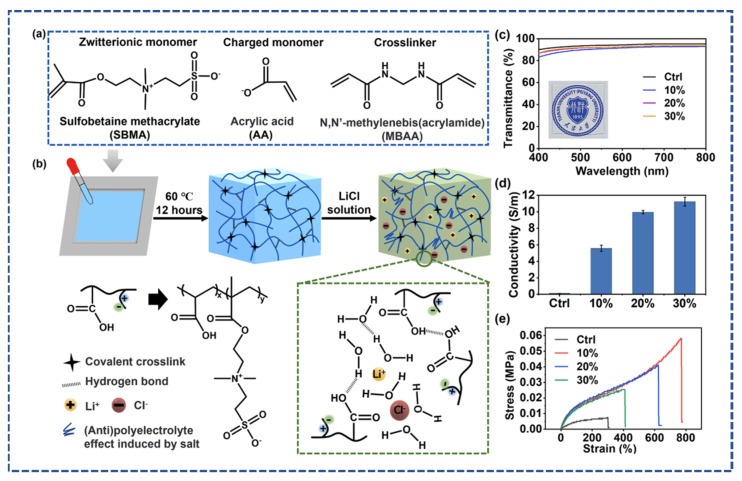
Synthesis of sulfobetaine-co-acrylic acid (SBMA-Co-AA) hydrogels. (**a**) Chemical structure of the crosslinker N, N′ -methylenebisacrylamide (MBAA), zwitterionic ionic monomer and charged monomer acrylic acid (AA) (**b**) Methodology of preparing the ionic conductive hydrogel (**c**) Transmittance graph with different concentration o of the samples (**d**) conductivity graph (**e**) Stress and strain graph under different concentration of LiCl solution reprinted with Copyright permission from ref. [46], 2021 Elsevier Ltd. (Amsterdam, The Netherlands).

**Figure 5:**
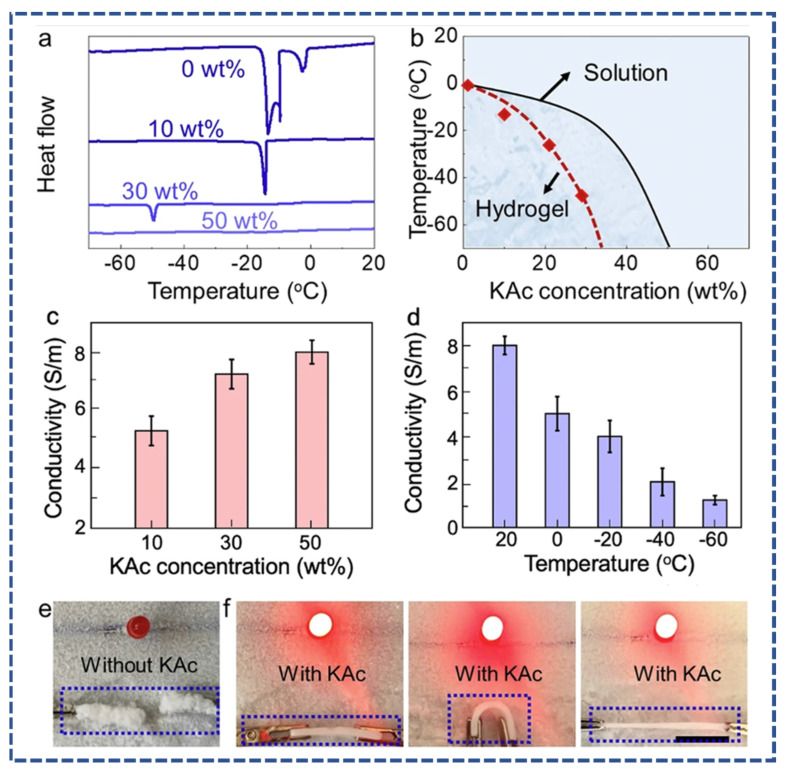
(**a**) Heat flow curves of hydrogel with varied concentration of potassium acetate (KAc), (**b**) transition temperature curve of the hydrogel treated with varied concentrations of potassium acetate (KAc) solution, (**c**) conductivity graph of the hydrogel when it’s treated with 10, 30, and 50 wt% concentrations of potassium acetate (KAc) solution, (**d**) conductivity graph of the hydrogel under varied temperature, and (**e**,**f**) visual anti-freezing testing of the conductive hydrogel reprinted from [47].

X. Sui et al. [46] reported on an innovative ionic conductive hydrogel containing (sulfobetaine-co-acrylic) acid. The results demonstrated excellent anti-freezing capabilities, which were tested under low temperatures (80 °C) for 30 days, and water retention qualities, which were confirmed under 25 °C, 54% humidity for 1 week and exhibited 100% retention of original water content. This work sets the stage for ionic hydrogels to operate throughout a wide temperature range. An overview of the preparation, transmittance, mechanical stress, and conductivity is shown in Figure 4. Wu et al. [47] investigated the effect of potassium acetate on polyvinyl alcohol to develop an anti-freezing, robust ionic conductive hydrogel, as shown in Figure 5. The proposed hydrogel exhibited high conductivity (8.0 S/m), tensile strength (8.2 MPa), and anti-freezing properties (−60 °C). Additionally, it showed excellent water retention and durability.

Overall, numerous findings are highlighted in the literature, and most studies demonstrated that materials with elastic mechanical properties, high sensitivity, and outstanding optical transparency provide promising prospects in wearable devices.

### 3.2. Electro-Conductive Hydrogels

Electroconductive hydrogel was reported by Guiseppi-Elie in 1995 [48], and later Wallace and Guiseppi-Elie stated that electroconductive hydrogels are networks of inherently conductive polymers that are highly hydrated [49]. Electroconductive hydrogels were created by combining conductive materials such as polypyrrole, polyaniline, and carbon nanotubes with conventional polymeric hydrogel chains [50,51]. Because of the combination of electroconductive and polymeric materials, the electro conductive hydrogel has a wide range of properties such as elastic mechanical properties, excellent optical properties, and good electrical conductivity [52].

**Figure 6:**
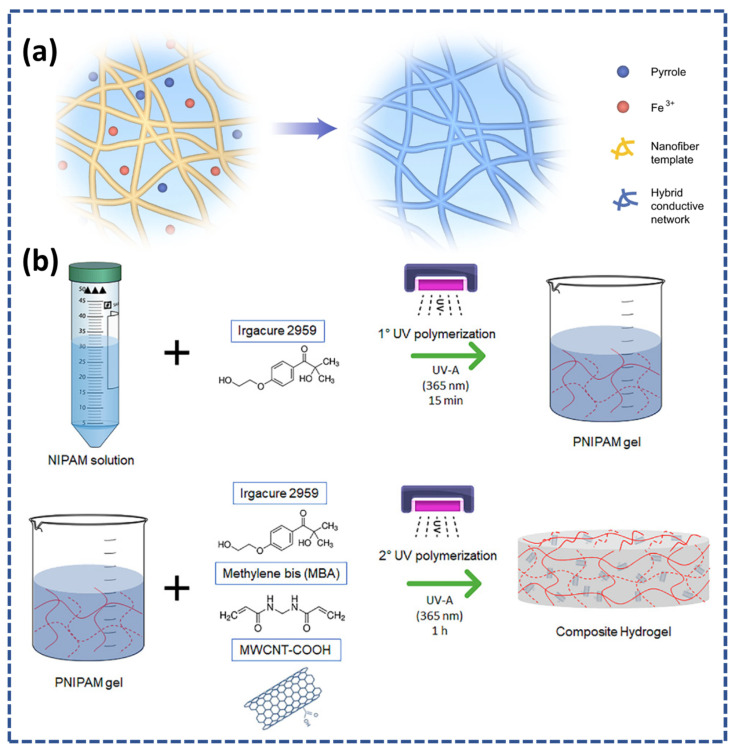
(**a**) Schematic of the synthesis of the conductive hydrogel from the polypyrrole, Fe^3+^, and hybrid conductive network reprinted with permission [53]. (**b**) Schematic of the two-step polymerization of the poly(N-isopropylacrylamide) matrix containing carboxyl-functionalized multi-walled carbon nanotubes (PNIPAM/MWCNT-COOH) hydrogel, reprinted from [54].

He et al. developed an innovative electroconductive hydrogel using polymeric nanofibers as shown in Figure 6a. The electroconductive hydrogel contains polypyrrole (PY), armid nanofibers, and polyvinyl alcohol (PVA). It displayed strong conductivity (80 S·cm), structural robustness, good mechanical strength (9.4 MPa), and fine stretchability (36%) without losing water content. The proposed electroconductive hydrogel can be used in electrophysiological applications [53]. Ciarleglio et al. reported the hybrid electro-conductive and thermosensitive hydrogel. The hybrid hydrogel was prepared from PNIPAM and multi-walled carbon nano tubes by two step polymerizations as shown in Figure 6b. The results demonstrated the enhanced sensitivity of hydrogel with excellent properties of electroconductivity and sensitivity [54]. Moreover, the literature highlighted that electroconductive hydrogels are also potential candidates for wearable bioelectronics.

### 3.3. Metal Based Conductive Hydrogels

Naturally, metals have excellent electroconductivity and outstanding mechanical properties. Due to their properties, researchers were attracted to integrating the hydrogel’s metallic particles to enhance their conductivity and mechanical properties [55].

Crosslinking between metallic particles and polymer chains is the main limitation of metallic-based conductive hydrogel [56]. Aside from that, cytotoxicity is a serious handicap. As a result, researchers are investigating various approaches to overcome this limitation, such as using modified metals including metallic nanoparticles, metallic wires, and nanotubes, to overcome cytotoxicity and crosslinking issues during synthesis [57].

## 4. Applications

Wearable technology has gained popularity recently due to its exceptional biocompatibility, flexibility, and accuracy. The world of material science is competing to invent new materials that are flexible and biocompatible to advance wearable technology [58].

Conductive hydrogels are the most suitable materials for wearable technologies due to their ease of synthesis, excellent conductivity, biocompatibility, and flexibility. Conductive hydrogels were used in biowearable technologies in a variety of ways, including motion sensors, strain sensors, and pH sensors, to monitor physiological parameters. Furthermore, conductive hydrogels were used as biowearable therapeutic systems [59].

### 4.1. Wearable Biosensing

Wearable biosensing is a demandable application of conductive hydrogels which includes strain sensing, motion sensing, electrochemical sensing, and biological sensing. In general, the strain concept is based on the effect of compression and stretch, which is clearly different from the strain sensor’s sensing mechanism. There are three types of flexible strain sensors: capacitive, resistive, and piezoelectric [60]. Each of the three types of sensors has its working principle, such as resistive sensors that convert stimuli into resistance changes, capacitive sensors that translate stimuli into capacitance, and piezoelectric sensors that detect the piezoelectric effect and output a potential difference [61].

**Figure 7:**
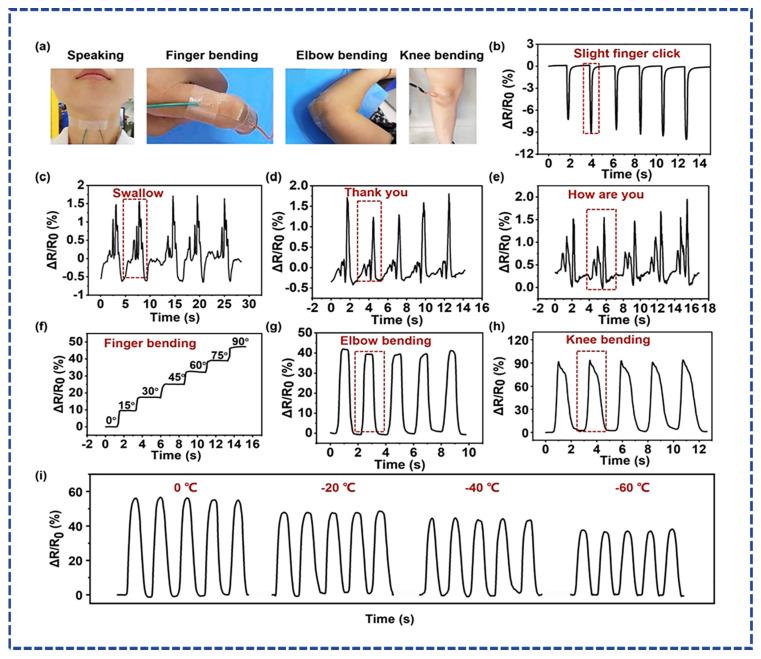
Real time motion detection. (**a**) Application of resistive sensor on the throat, finger, knee, and elbow. (**b**–**h**) Detected signal graphs of respective motions, such as finger click, swallowing, saying thank you, saying how are you, finger bending, elbow bending, knee bending. (**i**) Sensitivity over temperature range of (0–60 °C) reproduced from [46] with Copyright permission from, Elsevier Ltd.

In the context of conductive hydrogel-based strain sensors, most researchers design resistance-based strain sensors while fewer design capacitive strain sensors, and a negligible amount design piezoelectric strain sensors [62]. Liu et al. fabricated ferric cellulose nanocrystals and homogenous polymer-based soft ultrasensitive strain sensors. The prepared sensor demonstrated ultra-sensitivity, excellent stretchability, toughness, and mechanical strength. Additionally, the hydrogel contained the self-healing capability within 5 min without any external stimuli. The wearable strain sensor is applied for finger motion detection, breathing, and slight blood pulse detection [63]. Li et al. further tune the properties of the material by fabricating the conductive hydrogel from the Silver (Ag)/MXene nano networks and polyvinyl alcohol (PVA) borax matrix. The fabricated sensors reflected the high strain sensitivity with a gauge factor of (GF = 3.26) and self-healing within 10 min with antibacterial properties. The proposed material can be implemented in wearable monitoring biosensors [64]. Di et al. reported the highly conductive ionic PVA hydrogel synthesized by the salt solution soaking strategy. The prepared conductive hydrogel demonstrated outstanding tensile strength (8.03 MPa), elastic modulus (1 MPa), and toughness (28.7 MJ m^−3^). The sensitivity (7.14 S m^−1^) and accuracy (GF = 0.989) values demonstrated that the formulated hydrogel could be a promising candidate to use in wearable devices [65]. X. Sui et al. [46] reported the LiCL-based conductive hydrogel, which demonstrated excellent tensile strain with a gauge factor of (GF = 2.08). They fabricated the varied strain resistive sensor to apply in the real-time monitoring to fetch the physiological parameters. The fabricated versions applied for speaking motion, finger bending, knee bending and elbow bending, as shown in Figure 7a. From Figure 7b–h, the applied sensors detected the motions, such as speaking, finger, elbow, and knee motion. The sensor showed excellent biocompatibility over a wide temperature range (0–60 °C), as shown in Figure 7i. Overall, the results demonstrated the novelty of the proposed material.

**Figure 8:**
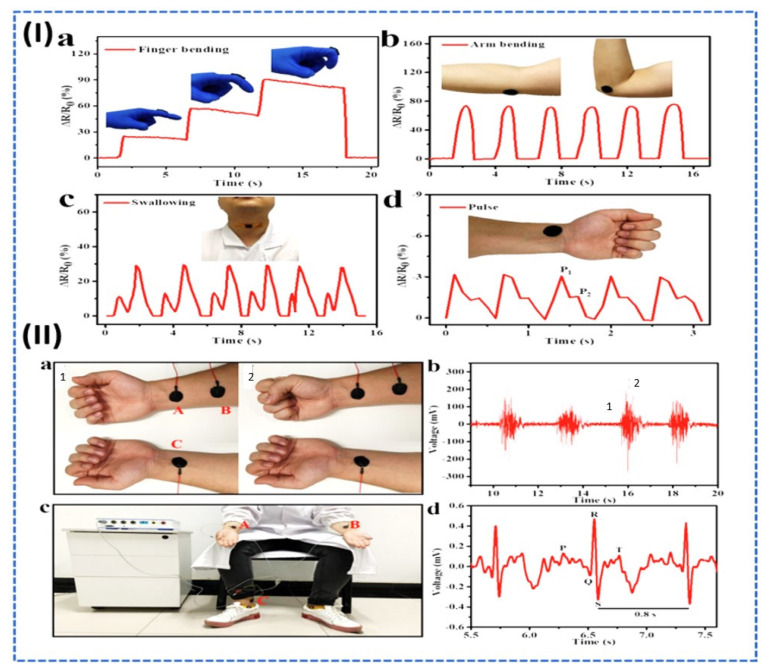
(**I**) Application of a sensor in motion detection. (**a**) Application of a sensor on the finger bending with observed motion signal. (**b**) Application of a sensor on the elbow with observed motion signal. (**c**) Application of a sensor on the throat with observed motion signal. (**d**) Application of a sensor in pulse wave detection with observed signal. (**II**) Application of a sensor as electronic skin. (**a**) EMG electrode application of the conductive hydrogel sensor, where A and B are the EMG differential electrodes and C is the reference electrode, also 1 and 2 show the hand movement. (**b**) Observed EMG signals, where 1 shows the relaxed hand position and 2 shows the closed hand position activity. (**c**) ECG setup based on the conductive hydrogel electrodes. (**d**) Observed ECG signal with P Q R S T wave with 97.4 beats/minutes from [66] with Copyright permission from American Chemical Society.

Li et al. reported the multifunctional conductive hydrogel for physiological monitoring based on the Mxene, PAA, and amorphous CaCO_3_. The reported hydrogel offered excellent stretchability, good self-healing, and high biocompatibility. Additionally, the proposed hydrogel was degradable and had high sensitivity with a fast response time of 20 ms as shown by Figure 8. It is also claimed as the electronic skin for physiological monitoring, such as ECG and EEG. Figure 8I highlighted the application of multiple functional conductive hydrogels in real-time motion detection. Figure 8(Ia) highlighted the resistance change with the respective bending angles (30°, 60°, and 90°), confirming the motion detection of the proposed conductive hydrogel-based sensor; while Figure 8(Ib) demonstrated the resistance change in the sensor with respect to the elbow bending. Figure 8(Ic) highlighted the motion detection of the throat during swallowing and Figure 8(Id) showed the alternate signals of the pulse to confirm the pulse detection application of the proposed hydrogel sensor. Furthermore, Figure 8II demonstrates the application of the Mxene and PAA as electronic skin to fetch electrophysiological signals, such as EEG and ECG. Figure 8(IIa) displayed the conductive hydrogel as an electrode on the arms of the object to fetch the EMG signals at different locations. Figure 8(IIb) displaced the observed signals of EMG in which I denoted the relaxing hand signal, and II denoted the contracted position signal. Furthermore, Figure 8(IIc) demonstrated the application of a sensor in the ECG setup, while Figure 8(IId) displaced the observed ECG signals [66].

Wang et al. invented a methodology to synthesis the conductive wearable sensor. The proposed methodology aims to solve the challenges the existing wearable biosensors face, such as low mechanical strength, poor stretchability, low adhesion properties, etc. The methodology applies to different materials, including 4-dihydroxy benzaldehyde, acrylamide, branched polyethyleneimine, poly (N-isopropyl acrylamide), LiCl, etc. The reported work could be used in the flexible wearable sensors to fetch the physiological signal of large and micro-movements of the human body during fracture rehabilitation [67]. Xiong et al. reported a new methodology to increase the conductivity and adhesiveness of the wearable sensor. The authors applied the methodology to the graphene material, synthesizing the conductive base liquid from sodium alginate, acrylamide and conductive nanofillers. The reported method consists of two steps. The first step synthesizes conductive nanofillers and flexible base liquids, while the second involves mixing, drying, ice bath, and cross-linking. Obtained graphene-based conductive hydrogel demonstrated excellent conductivity, self-healing, self-adhesiveness, and mechanical properties [68]. Roh et al. synthesized a new functional conductive hydrogel using a new innovative method in which authors used alginic acid, tannic acid, and albumins as main materials. The functional hydrogel properties varied according to the concentration of tannic acid. Furthermore, it demonstrated the outstanding properties of conductivity and flexibility and could be a potential conductive hydrogel for bio-wearable devices [69]. Dong et al. synthesized a transparent conductive hydrogel using a new technique for strain sensing. The reported hydrogel comprised anionic surfactant, methacrylic acid long-chain alkyl ester, initiators, and citric acid as cross-linkers. Moreover, it showed single-sided self-adhesion, transparency, large deformability, high mechanical strength, and excellent conductivity. Furthermore, it can bring about a revolution in bio-wearable devices and electronic skin [70]. Furthermore, the summarized potential investigations are listed in Table 5.

### 4.2. Wearable Therapeutic Patches

**Figure 9:**
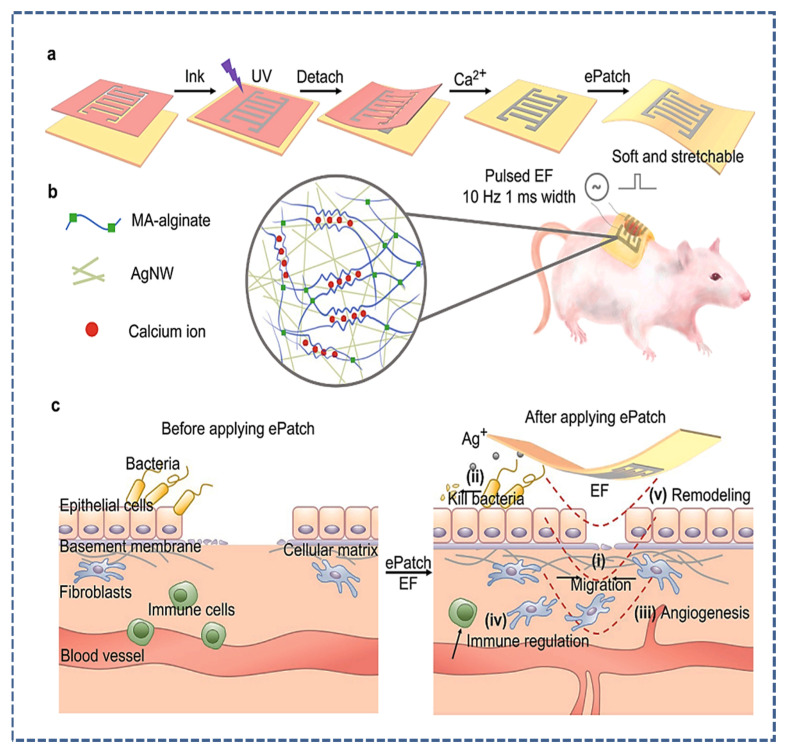
(**a**) Illustration of e-patch synthesis. (**b**) Schematic of hydrogel structure, component, and application on the mice model. (**c**) Illustration of the wound healing and biological activities at the wound site, reproduced from [110] with Copyright permission from, Elsevier Ltd.

Currently, wearable transdermal drug delivery systems are potential methods to minimize the side effects of traditional methods [108,109]. Various researchers implemented conductive materials in drug delivery systems to overcome the problems of sustained release and on-demand drug delivery. Among them, Wang et al. developed a wound-healing flexible electrical patch (epatch) composed of a conductive hydrogel (silver nanowires and methacrylated alginate), as shown in Figure 9. The e-patch demonstrated excellent wound closure, mediated immune response, outstanding angiogenesis, and antibacterial properties. Additionally, the in vitro results of the rat model showed wound closure within 7 days compared to 20 days, which is the usual healing period of rats. Figure 9a shows the synthesis mechanism of the epatch, Figure 9b demonstrates the overview of the conductive hydrogel components and epatch application on the mice model, Figure 9c highlights the mechanism of the wound closure before and after application of the epatch [110].

**Figure 10:**
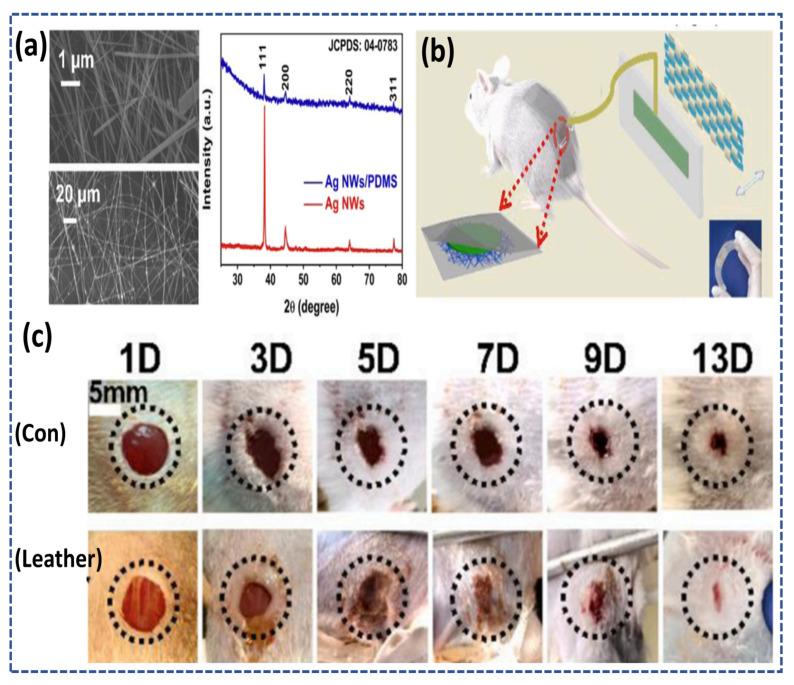
(**a**) SEM and EDX graphs of the silver nanowires (Ag-NWs) and silver nanowires/polydimethylsiloxane (Ag-NWs/PDMS) (**b**) Schematic of patch, and application on the mice model. (**c**) Practical photographs of wound healing reproduced from [111] with Copyright permission from Elsevier Ltd.

D. Wan et al. reported the wound healing conductive patch driven by the mechanical motion of the body. The patch was flexible, stretchable and based on the mechanism of triboelectricity. The conductive hydrogel acts as an electrode to mechanically transit motion-generated charges to the bottom layer, composed of silver nanowires to promote wound healing. Silver nanowires are also treated with other materials, such as polydimethylsiloxane (PDMS) to enhance the triboelectricity between the device and the human body, while the scanning electron microscopy (SEM) images and x-ray diffraction (XRD) data are shown in Figure 10a confirmed the successful compatibility between the silver nanowires (Ag-NWs) and polydimethylsiloxane (PDMS). Figure 10b highlights the illustration of the patch and its application in the rat model. Figure 10c demonstrated the visible wound healing of the leather group vs. the control group. It can be clearly understood from the pictures that the wound healing of the leather group was faster than the control group [111].

**Figure 11:**
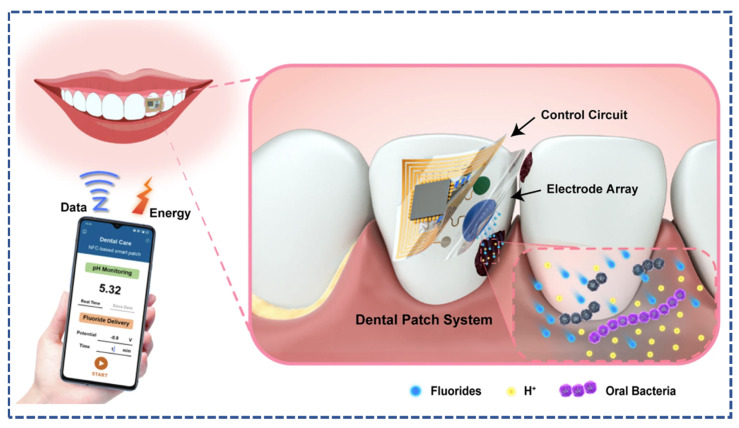
(Illustration of the wearable dental patch, wireless data control and application on the dental system reproduced from ref. [112] under open access creative common CC-BY license.

**Figure 12:**
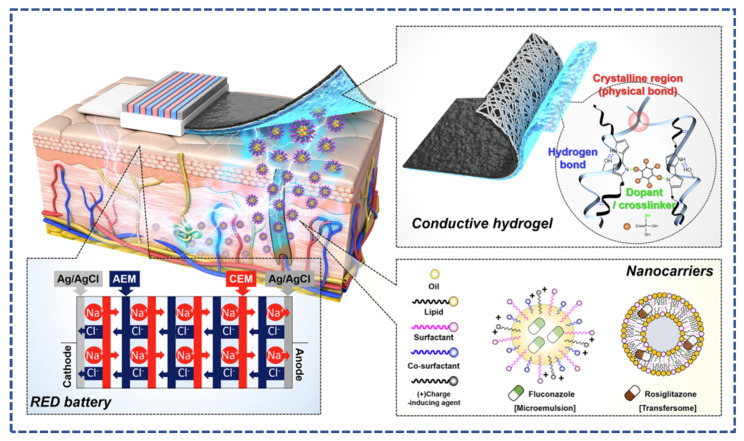
Illustration of transdermal iontophoretic system, structure of the conductive hydrogel and nanocarriers reproduced from [113] with Copyright permission from, American Chemical Society.

Z. Shi et al. developed a wearable, flexible patch for dental carries, a biocompatible, miniaturized and battery-free patch as shown in Figure 11 The patch contained electrosensitive electrodes, which delivered the fluorine drug based on the electrochemical detection of bacterial acidity. This work opens the door for a closed-loop drug delivery system based on conductive materials [112]. An et al. developed the transdermal iontophoretic drug delivery system based on the reverse electrodialysis battery and delivered the therapeutics through the ion exchange phenomenon, as shown in Figure 12. The electroconductive system of the device was made up of poly (vinyl alcohol) and polypyrrole; additionally, the charged drug nanocarriers were used as delivery agents. The proposed iontophoretic system offered an effective application for antiobesity conditions. Figure 12 illustrates the transdermal iontophoretic system, reverse electrodialysis battery and chemical structure of the nanocarriers [113].

Xiong et al. disclosed a new synthesis technique of conductive material to treat cartilage. The reported method was applied to the cellulose chains, where dopamine was inserted to destroy the hydrogen bonds between cellulose chains to enhance the toughness. At the same time, for surface modification, graphene was used. The synergetic effect of the polydopamine and poly graphene oxide improved the enhanced conductivity and mechanical properties. The reported work could be used as a potential candidate for artificial skin or cartilage repair [114]. Jianyong et al. disclosed a new method to measure cell impedance by synthesizing conductive hydrogel. The conductive hydrogel comprised the conductive microchip, which had microelectrode arrays and a cell culture chamber. The micropattern on the microchip is made using the electrochemical deposition method. So, the disclosed invention could replace the metal electrode system for dynamic and real-time analysis of impedance sensing systems [115]. Perez et al. disclosed a new electro-dermal patch to treat dysmenorrhea and its symptoms. The reported device comprised the microprocessor, electrical stimulator, and electrode system. The electrode probe delivered electrical stimulation to the patient’s epidermal layer in the range of 0.1mm to 20mm. Additionally, the device communicated wirelessly with a control device to monitor and record the patient’s status [116]. Verbeck et al. disclosed an invention to transport pharmaceutical agents, nutraceuticals, and electrolytes via the skin or trans mucous membranes using the reported technique and material. The invention provides products of manufacture that are composed of controlled melt or solubilization of polymer coupled with the nanoporous substrate to deliver the payloads at targeted regions. The reported work could be a potential technique in transdermal drug delivery systems [117]. Boggs et al. reported a new implantable device for locating the tissue region. The device comprised the inner sheath, which contained the implantable electrode, while the outer sheath was coupled with the power source and simulating signal circuitry. The professionals controlled the simulated signal to tissue regions via the outer sheath. Therefore, this device opened the door for professionals to locate the exact regions of the affected tissues [118].

## 5. Summary and Future Direction

The literature shows the tremendous interest of researchers in developing conductive hydrogels due to their excellent flexibility, biocompatibility, and conductivity in wearable bioelectronics. Conductive hydrogel has many benefits over traditional sensing and therapeutics materials due to flexibility and biocompatibility. Current development of conductive hydrogel includes smart transdermal drug delivery systems, hydrogel-based smart batteries, smart electrodes for enhanced bioelectronics, and smart medical imaging systems.

Wearable bioelectronics is one of the most exciting areas in which researchers are attracted worldwide due to the community demand because of their low weight, high deformability, high accuracy, high flexibility, and time-saving advantages. Researchers are researching novel ways to fabricate complicated and biomedically valuable hydrogel-based wearable bioelectronics.

However, some limitations are still associated with conductive hydrogels in wearable bioelectronics. One of the challenges is the difficulty in fabricating conductive hydrogel sensors that have biocompatibility, antibacterial properties, and toughness. Another limitation is the performance of the hydrogel-based devices, which are strongly influenced by the type of conductive components used, such as ionic and electronic conductors, carbon-based, metal-based, or conductive polymer-based. Furthermore, the input energy sources, input/output range, and power consumption of the IC must be carefully considered when designing self-powered sensors that can store harvested energy in an energy buffer, normally a supercapacitor or a rechargeable battery.

The future of conductive hydrogels in wearable bioelectronics looks bright, with ongoing research focused on developing soft, biocompatible conductive hydrogels with low modulus and high electrical conductivity. Natural biopolymer conductive hydrogels have been identified as promising materials for flexible wearable sensors and energy devices, with recent progress in their development. As research continues, we can expect to see more innovative applications of conductive hydrogels in the wearable bioelectronics industry.

## Figures and Tables

**Table 1:** Summarized Overview of Conductive Polymers.

Polymer	Conductivity (S·cm^−1^)	Modulus of Elasticity (GPa)	Advantages	Disadvantages	Applications
PPY [18,21]	10~50	0.00800~8.25	Strong mechanical properties, Flexible and Biocompatible	Lack of mechanical stability after doping and poor thermal properties	Bioelectronics, Biosensors, and biotherapeutics
PANI [19]	10^−1^~10^5^	0.05~1	High sensitivity and reversible doping	Low conductivity and poor chemical stability	Biosensors, bio actuators, and drug delivery patches
PEDOT [22]	3 × 10^2^~5 × 10^2^	0.5~2.8	Excellent optical transparency, simple doping, chemical stability	Low mechanical stability and corrodes metal materials.	Biosensors, close loop drug delivery patches, and tissue engineering
PAT [23]	100	0.03~1	Strong mechanical property and excellent thermal properties	Low electrochemical properties and low solubility	Biosensors and tissue engineering
PTH [24]	57.2	0.03~12	High conductivity and excellent thermal and chemical stability	Low solubility and complex doping	Electrodes, actuators, and electronic material
PPV [25]	0.001~100	0.03~50	Better solubility and good thermal properties	Low electroluminescent and fluorescence quenching	Photovoltaic device, transistors, biosensors, and actuator

**Table 3:** Summarized Properties of Carbon derivatives.

Carbon Derivatives	Diameter (nm)	Density (g/cm^3^)	Conductivity (S·cm^−1^)	Advantages	Limitations	Applications
Carbon NPs [34]	~2–100	~2.26	1~10^4^	High conductivity and sensitivity	Long term cytotoxicity	Anticancer, drug delivery, and biosensing
Carbon Nanotubes [35]	~0.4–40	~1.4	10^2^~10^6^	High thermal properties and Conductivity	Insolubility and non-uniformity	Energy storage devices, coating, and actuators
Carbon dots [36]	~2–4	~1.032	10^1^~10^8^	High conductivity and luminance properties	Complex synthesis process	Actuators, batteries, and biosensors

**Table 4:** Summarized Properties of Hybrid Conductive materials.

Hybrid Material	Conductivity (S·cm^−1^)	Advantages	Limitations	Applications
PEDOT: PSS [39]	867	High conductivity, high flexibility, sensitivity	Acidity, hygroscopicity	Wearable electronics, molecular sensing, and biosensing
Poly acrylamide and PEDOT: PSS [40]	200	High Conductivity, transparent, and high thermal properties	Acidity and non-uniformity	Wearable electronics and biosensing
PPAM: PEDOT: PSS [44]	6.0 × 10^−2^	High conductivity and good optical properties	Poor self-adhesion	Biosensors and wearable electronics.

**Table 5:** Summarized potential works on biosensing.

Material-Conductive Hydrogel	Application	Authors
N-acryloyl phenylalanine, acrylic acid, ferric chloride	Wearable Electronics	Shen et al., 2023 [71]
Poly(N-isopropylacrylamide) PNIPAm, sodium dodecyl sulfate (SDS)	Wearable Iontronics	Bai et al., 2023 [72]
Agar/Borax/MXene	Flexible sensors	Nie et al., 2023 [73]
PAM, SA and LiCl	Flexible sensors	Zhang et al., 2023 [74]
Lauryl methacrylate acrylamide sodium alginate	Wearable Sensos	Yazdani et al., 2023 [75]
polyacrylic acid/polyvinyl alcohol (PAA/PVA) (choline chloride, glycerol, Lewis’s acid	Flexible Sensors	Yan et al., 2023 [76]
Hyaluronic acid	electro bio sensing	Aycan et al., 2023 [77]
HA-DA-PP	electro bio sensing	Zang et al., 2023 [78]
Graphite, zwitterionic monomers	Bioelectronics	I.k et al., 2023 [79]
Poly(ACMO)/Pt	Flexible Sensors	Guo et al., 2023 [80]
Agarose PEDOT: PSS	Tissue engineering	Casella et al., 2023 [81]
Metal liquid and CNT	Flexible Sensors	Sun et al., 2023 [82]
Liquid metal, Mxene, Bacterial cellulose	electro biosensing	Wang et al., 2023 [83]
PHEMA/TA-Fe	Wearable biosensing	F. Wang et al., 2023 [84]
UPAM-Mxene-LM	Wearable biosensing	Dong et al., 2023 [85]
Poly(amidoxime)/polyethyleneimine (PAO/PEI)	Flexible Sensors	Xu et al., 2023 [86]
Acrylic acid, 1-vinyl-3-butylimidazolium bromide and aluminum ion	Biosensing	Zhou et al., 2022 [87]
Sulfonated lignin-coated silica nanoparticles (LSNs), polyacrylamide (PAM) chains, and ferric ions	Biosensing	H. Zhou et al., 2022 [88]
Polypyrrole (PPy) silk fibroin (SF) and tannic acid (TA)	Strain sensing	Zheng et al., 2022 [89]
PVA/gelatin/β-CD	Strain sensing	Fan et al., 2022 [90]
Lignosulfonate/polyvinyl alcohol and silver	Strain sensing	Wu et al., 2022 [91]
PAANa/PEDOT: PSS/PVA	Flexible sensing	Gong et al., 2022 [92]
Polyvinyl alcohol (PVA) and polyaniline (PANI)	Strain sensing	Sun et al., 2022 [93]
Graphene oxide, polyvinyl alcohol-polyacrylamide	Biosensing	Dai et al., 2022 [94]
CMC/PAA/Fe^3+^/LiCl	Strain sensing	Song et al., 2022 [95]
Polypyrrole, Silk	Strain sensing	Han et al., 2022 [96]
Cellulose/phytic acid/polyaniline	Strain sensing	Wan et at. 2022 [97]
PVA and cellulose nano fibers	Strain Sensing	Wu et al., 2022 [98]
Polyacrylamide, lithium magnesium silicate, carbon quantum dots	Strain Sensing	Yu et al., 2022 [99]
Acrylic acid, acrylamide, 2-methacryloyloxyethyl phosphorylcholine, chitosan	Strain Sensing	Chen et al., 2022 [100]
Mxene, polyvinyl alcohol/sodium carboxymethylcellulose, tannic acid	Biosensing, Strain Sensing	Kong et al., 2022 [101]
PVA/SA/Mxene	Biosensing, Strain Sensing	Wang et al., 2022 [102]
Amylose, polyvinyl alcohol, glycerol/NaCl	Biosensing	Gao et al., 2022 [103]
gelatin/NaCl organo hydrogel	Biosensing, Strain Sensing	Wu et al., 2022 [104]
Polyvinyl alcohol, polyethylene glycol, chitin nanocrystals	Strain sensing	Cai et al., 2022 [105]
Polyacrylamide, gelatin, polyurethane	Biosensing, Strain Sensing	Wang et al., 2022 [106]
Sodium alginate, polyacrylamide, silica, carbon nanotubes	Flexible Sensors	Zhang et al., 2022 [107]

## Data Availability

Not applicable.

## Abbreviations

PPV	Poly(p-phenylene vinylene)
PPY	poly pyrrole
PVA	poly(vinyl acetate)
PDOT	poly-(3,4-ethylenedioxythiophene)
PNIPAm	Poly(N-isopropylacrylamide)
PAT	Polyacetylene
PTH	Polythiophene
NPs	Nano Particles
β-CD	Beta Cyclodextrin
PAA	Polyacrylic acid
PAM	Polyacrylamide
PHEMA	Poly(2-hydroxyethyl methacrylate)
UPAM	U-polyacrylamide
HA	Hyaluronic acid
PANI	Poly-(3,4-ethylenedioxythiophene
SF	Silk fibroin
TA	Tannic acid
LSNs	sulfonated lignin-coated silica nanoparticles
Pt NPs	Platinium Nanoparticles
Au NPs	Gold Nano Particles
Pd NPs	Pladdinium Nano Particles
Ag NPs	Silver Nanoparticles
SA	Sodium Alginate
